# Effect of Sertraline on Fetoplacental Growth Parameters and Placental Transporter Gene Expression in Rats

**DOI:** 10.3390/ijms27093858

**Published:** 2026-04-27

**Authors:** Daniel Enriquez-Mendiola, Jorge E. Sifuentes-García, Laura J. Barragán-Zúñiga, Angel A. Vértiz-Hernández, Blanca P. Lazalde-Ramos, Alicia E. Damiano, Carlos Galaviz-Hernández, Martha Sosa-Macías

**Affiliations:** 1 Genomics Academy, CIIDIR-Durango, Instituto Politécnico Nacional, Durango 34220, Mexico; qfb.dan.enriquez@hotmail.com (D.E.-M.); jorgej2318@gmail.com (J.E.S.-G.); 2 Centro Estatal de Cancerología, Secretaria de Salud de Durango, Durango 34000, Mexico; ljbarraganz@gmail.com; 3 Unidad Académica Regional Altiplano, Universidad Autónoma de San Luis Potosí, San Luis Potosí 78600, Mexico; antonio.vertiz@uaslp.mx; 4 Maestría en Ciencia y Tecnología Química, Unidad Académica de Ciencias Químicas, Universidad Autónoma de Zacatecas, Zacatecas 98160, Mexico; lazalderamos@uaz.edu.mx; 5 Departamento de Ciencias Biológicas, Facultad de Farmacia y Bioquímica, Universidad de Buenos Aires, Buenos Aires C1113AAD, Argentina; aedamiano@gmail.com; 6 Instituto de Fisiología y Biofísica Bernardo Houssay, Universidad de Buenos Aires-CONICET, Buenos Aires C1121ABG, Argentina

**Keywords:** SSRI, fetal development, placental efficiency, drug transporter, nutrient transporter

## Abstract

The aim of this study was to assess the effect of sertraline on the gene expression of placental transporters for hormones, folates, nutrients and drugs over the course of pregnancy in rats. The studies were conducted on gestational days (GDs) 16 and 20 following oral treatment with 10 mg/kg/day sertraline or the vehicle, administered from weaning onward. The weight and area of the fetuses and placentas were analyzed, and maternal plasma sertraline concentrations were measured. Gene expression of ATP-binding cassette transporter b1a and b1b (*Abcb1a* and *Abcb1b*), organic anion-transporting polypeptide 4a1(*Slco4A1*/*Oatp4a1*), folate receptor-α (*Folr1*), reduced folate carrier (*Slc19A1*/*Rfc*), and L-type amino acid transporter (*Slc7A5*/*Lat1*) was evaluated in the placenta. Sertraline reduced fetal weight (*p* < 0.001) and fetal area (*p* < 0.01) at GD 16, while no significant differences were observed in placental weight or area between exposed and unexposed groups. Sertraline concentration was significantly lower at GD20 than at GD16 (*p* < 0.001). At GD 16, sertraline reduced the expression of *Abcb1a* (*p* = 0.027), *Abcb1b* (*p* < 0.01), and *Oatp4a1* (*p* = 0.037) compared with controls. Conversely, sertraline induced *Folr1* expression in both GDs and increased *Rfc* expression at GD 20, while *Lat1* was not affected. These findings indicate that sertraline alters placental drug transporter gene expression and may impair nutrient transfer to the fetus.

## 1. Introduction

Sertraline is one of the most prescribed selective serotonin reuptake inhibitors (SSRIs) for use during pregnancy [1]. It is considered safer than tricyclic antidepressants and is not associated with an increased risk of teratogenicity compared with no treatment [2]. However, chronic exposure to sertraline before and during early pregnancy has been associated with a range of adverse outcomes in offspring, including omphalocele, limb defects, anencephaly, anal atresia, congenital heart defects, pulmonary hypertension, an increased risk of autism in males, low birth weight, and preterm birth [3]. The mechanisms underlying these effects remain poorly understood but may involve the extent of exposure to xenobiotics, as well as alterations in transplacental nutrient transport, which could negatively impact the intrauterine environment and fetal development.

The placenta expresses several transport proteins specialized in the exchange of nutrients, the removal of metabolic waste products, and the transfer of exogenous compounds between the mother and the fetus. These transporters are localized to either the maternal-facing (microvillous membrane, MVM) or the fetal-facing (basal plasma membrane, BM) side of the placenta and are classified into two major groups: ATP-binding cassette (ABC) transporters and solute carrier (SLC) transporters [4].

The transporter P-glycoprotein (P-gp), also known as ABCB1, is one of the predominant efflux pumps distributed in the MVM and its substrates include a wide range of xenobiotics [5]. Hence, the primary function of ABCB1 is to prevent substances in maternal blood from entering the fetal compartment. ABCB1 is encoded by the *ABCB1* gene in humans and by *Abcb1a* and *Abcb1b* genes in rodents, being highly homologous to human P-gp (80%) [6].

The L-type amino acid transporter LAT1 (*SLC7A5*) and the organic anion transporting polypeptide OATP4a1 (*SLCO4A1*) are abundantly expressed in the MVM and play an important role in the transport of thyroid hormones [7]. In addition, LAT1 performs the exchange of neutral aromatic and long-branched chain amino acids with 1:1 stoichiometry [8]. Other relevant proteins, such as folate carriers including folate receptor-α (FRα/*FOLR1*) and reduced folate carrier (RFC/*SLC19A1*), contribute to the influx of folate in the placenta [9]. FRα is localized to the MVM and RFC in the MVM and BM [10]. Several studies have demonstrated that medications administered during pregnancy, particularly antiepileptic drugs, modulate the placental expression of these types of transporters [11,12,13,14,15]. The effect of antidepressants such as SSRIs in pregnancy on the expression of placental transporters has been partially studied. Among these, sertraline and other SSRIs modulate serotonin levels in the placenta and fetus by inhibiting the SERT and OCT3 transporters, as demonstrated in in vitro and in situ models [16], suggesting that other placental transporters can also be affected. Based on this, we hypothesized that the administration of sertraline during pregnancy may affect both the placental expression of *Abcb1a*, *Abcb1b*, *Rfc*, *Folr1*, *Lat1*, and *Oatp4a1*, as well as the somatometry of the placenta and fetus.

Therefore, the aim of this study was to evaluate placental and fetal morphometric changes and the gene expression of the placental transporters *Abcb1a*, *Abcb1b*, *Rfc*, *Folr1*, *Lat1*, and *Oatp4a1* in response to chronic sertraline administration in pregnant Wistar rats in two different gestation stages. This study was designed as an exploratory approach to characterize gestational stage-dependent changes in placental transporter gene expression following sertraline exposure.

## 2. Results

### 2.1. Number of Fetuses and Fetal and Placental Somatometry

**Figure 1:**
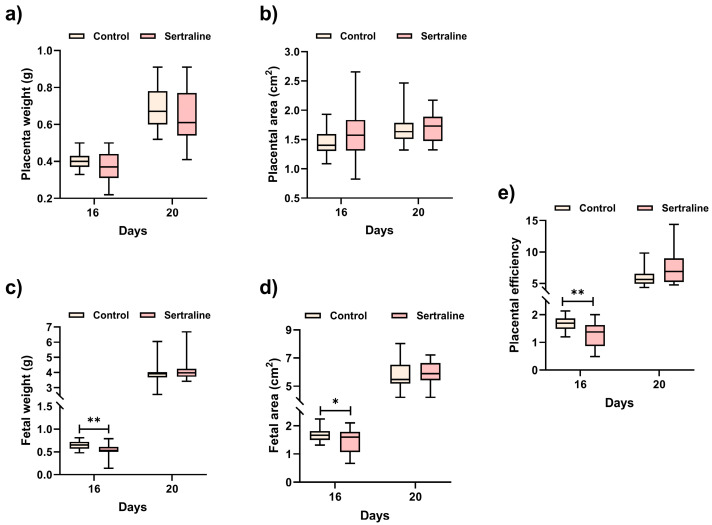
Somatometry of placentas and fetuses from pregnant rats exposed to sertraline or distilled water (H_2_O) as a control on gestational days 16 and 20. The effect of sertraline on the (**a**) weight and (**b**) area of placentas, (**c**) the weight and (**d**) area of fetuses, and (**e**) placental efficiency (the fetal-to-placental weight ratio). Samples: control (n = 6) and sertraline (n = 6). The results are illustrated as median, minimum, and maximum values. The comparisons between the study groups were made using the Mann–Whitney U test, where * *p* < 0.05 and ** *p* < 0.001.

A total of six placentas and six fetuses per stage were evaluated. We characterized placental and fetal somatometric parameters in our study groups. No significant differences in the number of fetuses on any GDs were found between the exposed and non-exposed groups. No statistically significant differences were detected in placental weight and area between control placentas and those exposed to sertraline (Figure 1a,b).

Regarding morphometric analyses of the fetuses, on GD 16, the fetuses exposed to sertraline presented a significantly lower weight (*p* < 0.001) and area (*p* < 0.01) compared with the control group. These differences were ablated on GD 20 (Figure 1c,d). None of the fetuses showed any major congenital malformations.

Placental efficiency was evaluated on GD 16 and GD 20. A significant decrease in placental efficiency was noted in the exposed group on GD 16 (*p* = 0.002). No differences in placental efficiency between the exposed and non-exposed groups were observed on GD20 (*p* = 0.051) (Figure 1e).

### 2.2. Maternal Sertraline Plasmatic Concentrations

**Figure 2:**
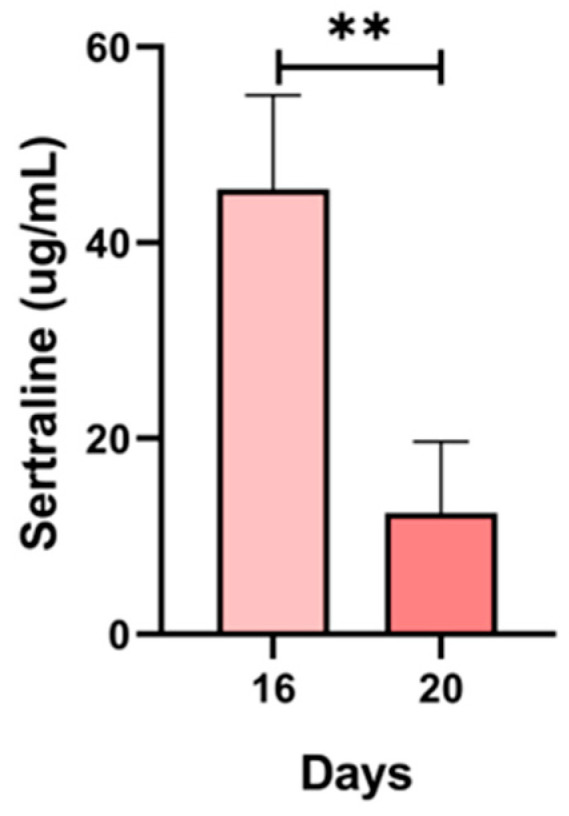
Sertraline concentration in the plasma of pregnant rats on gestational days (GDs) 16 and 20. Samples: n = 6 per GD. The results are presented as the mean ± SD. Comparisons between the exposed groups were performed using analysis of variance (ANOVA) with post hoc Tukey’s test, where ** *p* < 0.001.

The presence of sertraline was confirmed in the plasma of pregnant animals in the treated group (n = 6). The concentration of sertraline showed a statistically significant decrease on GD 20 compared with those found on GD 16 (*p* < 0.001) (Figure 2).

### 2.3. Relative Gene Expression

**Figure 3:**
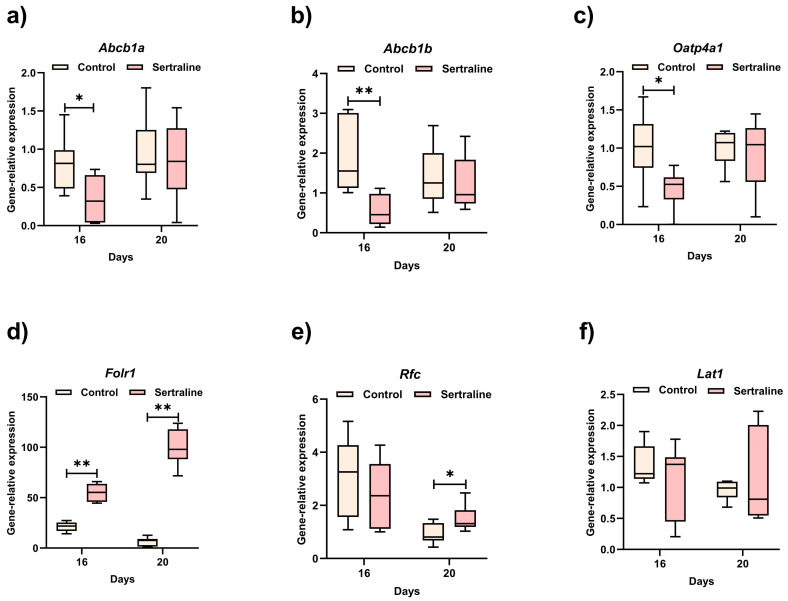
The effect of sertraline administration on the expression of (**a**) *Abcb1a*: ATP-binding cassette transporter, (**b**) *Abcb1b*, (**c**) *Oatp4a1*: organic anion-transporting polypeptide 4a1, (**d**) *Folr1* or *Fr-α*: folate receptor α, (**e**) *Rfc:* reduced folate carrier, (**f**) *Lat1*: L-type amino acid transporter 1, and mRNA in rat placentas on gestational days 16 and 20. The results presented are median, minimum, and maximum values from 6 placentas (in duplicate) in each experimental group. Study groups were compared using the Mann–Whitney U test, where * *p* < 0.05 and ** *p* < 0.01.

The comparative gene expression of transporters of six placentas in each experimental group on GDs 16 and 20 is shown in Figure 3. On GD 16, the sertraline-exposed group showed a significant decrease in *Abcb1a* (*p* = 0.027), *Abcb1b* (*p* = 0.003), and *Oatp4a1* (*p* = 0.037) compared with the non-exposed group; conversely, the expression of *Folr1* was significantly higher in the exposed group (*p* = 0.006). No differences between groups were observed for *Rfc* and *Lat1*. On GD 20, overexpression of *Folr1* (*p* = 0.002) and *Rfc* (*p* = 0.046) was observed in the exposed group; meanwhile, the remaining transporters showed no significant differences.

## 3. Discussion

We report the effects of sertraline on placental and fetal somatometric parameters, placental efficiency, and gene expression of rat placental transporters. Placental efficiency was assessed using weight-based indices and should be interpreted as a gross morphological parameter rather than a direct functional measure. These transcriptional changes should not be interpreted as direct evidence of altered transporter activity or placental efficiency, but rather as gene expression-level associations within the scope of this exploratory study. The dose of sertraline used in our study (10 mg/kg/day) is equivalent to a dose of 100 mg/day administered to humans and corresponds to the treatment commonly followed in pregnancy, which is considered clinically safe [3]. Our results revealed no sertraline-induced changes in placental weight and area on GDs 16 and 20. Although fetal weight and area were smaller in the sertraline-treated group on GD 16 (*p* < 0.01), these alterations were no longer observed on GD 20. Although we did not investigate the underlying mechanisms, these alterations in fetal morphometry may be associated with sertraline.

This drug, like other SSRIs, increases maternal circulating serotonin, augmenting vasoconstriction of the uterine and placental vascular beds [17,18]. This event decreases blood perfusion, affecting fetal growth and development [19,20]. In addition, previous research has reported that hypoxia causes significant changes in the placenta, augmenting the placental size relative to birth weight [21]. This report agrees with the significant decrease in placental efficiency (*p* = 0.002) observed on GD 16 in this study, but not on GD 20. The consistent overexpression of the *Folr1* transporter, together with the overexpression of *Rfc* and the recovery of the expression levels (like those of the control groups) of the remaining transporters (*Abcb1a*, *Abcb1b*, and *Oatp4a1*) by GD 20, may compensate for metabolic requirements, thereby supporting the viability of the fetuses. The significant drop in the concentrations of sertraline on GD 20 could explain why fetal alterations were resolved at the end of gestation.

Human newborns exposed prenatally to sertraline at doses up to 100 mg/day showed no differences in weight or size compared with unexposed newborns [22]. In a rodent model, at a dose of 5 mg/kg/day of sertraline, no differences in fetal weight were observed between the control groups and the exposed groups; however, supporting our findings, fetal weight was significantly lower in the groups exposed to 25 and 60 mg/kg/day [23]. In the same study, the authors observed visceral and skeletal abnormalities at the highest doses of sertraline (25 and 60 mg/kg/day) that were not observed at the lowest dose (5 mg/kg/day) [23]. We did not observe malformations in any of the fetuses exposed to sertraline. The same result was observed by Pawlusky et al. (2020) [24], who did not find fetal abnormalities with sertraline doses of 2.5 and 10 mg/kg/day in pregnant rats. Despite these findings, a potential teratogenic effect of sertraline at higher doses cannot be completely ruled out.

The sertraline concentration in maternal plasma dropped on GD 20. This finding coincides with previous observations in pregnant women revealing a decrease in sertraline concentration during the last half of gestation [25]. These reduced levels of sertraline towards the end of pregnancy are attributed to the increase in drug metabolism by different CYP450 enzymes [25]. The underlying mechanisms of CYP450 upregulation have been associated with hormonal dynamics during pregnancy [26]. Additionally, the decrease in sertraline concentrations could also be related to the increase in the volume of distribution (Vd) and the elimination rate towards the end of gestation [26]. In this regard, the Vd during pregnancy increases progressively because of an approximate 50% expansion of plasma volume [27]. This increase in Vd is associated with a reduction in the maximum serum concentration (Cmax) of many drugs. In addition, the pregnancy-associated increase in adipose tissue stores contributes to a larger Vd for lipophilic drugs, which may further reduce their peak plasma concentrations. Furthermore, the increase in cardiac output during gestation leads to enhanced hepatic blood flow, which may result in more rapid elimination of high-extraction drugs such as sertraline [28].

In this study, sertraline was quantified as total plasma concentrations without distinguishing between free and plasma protein-bound fractions. This is relevant given sertraline’s high plasma protein binding capacity (~98%), primarily to albumin and α1-acid glycoprotein [29], indicating that only a small unbound fraction is available for placental transfer. Consequently, total concentration measurements may overestimate the fraction potentially transferable to the fetal compartment. Nevertheless, total plasma concentrations remain informative, as they reflect overall maternal systemic exposure and allow for comparison with existing preclinical and clinical data. However, future studies should consider measuring the unbound fraction to more accurately assess placental transfer and fetal exposure.

The efflux transporter of drugs P-glycoprotein (P-gp), also known as ABCB1, is encoded by the *ABCB1* gene in humans and by *Abcb1a* and *Abcb1b* genes in rodents, being highly homologous to human P-gp (80%) [6]. As noted above, these transcriptional changes should not be interpreted as direct evidence of altered transporter activity or placental efficiency. Although *Abcb1a* and *Abcb1b* have been detected in the placenta, the former has predominant expression in the blood–brain barrier (BBB) [30]. In the present study, the expressions of *Abcb1a* and *Abcb1b* genes decreased significantly on GD 16 in the exposed group; meanwhile, no changes were observed on GD 20. This sertraline-induced decrease in *Abcb1a* and *Abcb1b* gene expression may reflect transcriptional modulation of placental *Abcb* transporters, rather than a direct reduction in protein expression or transporter activity. Previous studies have described an accumulation of P-gp substrates at the BBB following reduced efflux in maternal and fetal compartments because of exposure to sertraline [31]. In another study, sertraline had a dual time-dependent effect on the activity of P-gp (inhibition at 5 and 240 min and stimulation at 60 min) at the BBB and the blood–testis barrier [32]. Additionally, an inhibitory effect of the sertraline metabolite desmethylsertraline on P-gp has been reported in L-MDR1 cells (model for human ABCB1) and primary porcine brain capillary endothelial cells (model for blood–brain barrier) [32,33]. In this context, the accumulation of desmethylsertraline on GD 16 may represent a possible contributing factor; however, this interpretation remains speculative and requires confirmation through protein expression and functional assays. Finally, on GD 20, the expressions of *Abcb1a* and *Abcb1b* in the sertraline-exposed and non-exposed groups were similar, which might be explained by the decrease in sertraline concentration at term.

The Oatp4a1 transporter has a high affinity for thyroid hormones (THs) [7] and is one of the most abundant Oatps expressed in the placenta [34]; therefore, it could play a key role in the transport of THs from maternal blood across the placenta. It has been reported that mRNA *Oatp4a1* increased throughout gestation [13,34], which is concordant with the results of this study: the control group displayed an increase in the expression of *Oatp4a1*, while the exposed group showed a decrease on GD 16 (*p* = 0.037). The reduced expression of *Oatp4a1* may be associated with decreased TH supply to the placenta and fetus, potentially contributing to the reduced fetal weight observed on GD 16. However, this association does not establish causality, and functional validation is required to confirm the effects on TH transport. Prior studies have shown that in small for gestational age (SGA) fetuses, serum levels of thyroid-stimulating hormone (TSH) are significantly higher and levels of T4 and free T4 are significantly lower compared with appropriate for gestational age (AGA) fetuses [35]. Similar findings have been reported for serum concentrations of free T4 and free T3, which were found to be lower in fetuses affected by intrauterine growth restriction (IUGR), although serum TSH levels were not significantly different [36]. In addition, the impact of gestational exposure to SSRIs on thyroid function in newborns has previously been evaluated. A clinical study including 21 women who received SSRIs during their entire pregnancy and 20 healthy pregnant controls showed that the SSRI group had significantly higher levels of cord blood TSH (*p* < 0.004) and a significantly higher percentage of newborns in the SSRI group had TSH levels > 15 ng/mL (38% vs. 5%, *p* < 0.001) compared with the controls [37]. The SSRI group was also characterized by a significantly elevated incidence of birth weight below the 10th percentile for gestational age. In contrast, a large-scale study with 2321 mothers treated with SSRIs during pregnancy and 103,607 controls reported that TSH and total thyroxine (TT4) levels in newborns did not differ between the SSRI group and the general population, although a greater proportion of newborns in the SSRI group required TSH testing due to a relatively low TT4 level (14% vs. 9%, *p* < 0.001) [38]. In the present study, fetal weight was restored on GD 20, the same point at which *Oatp4a1* expression levels were found to be similar between the sertraline-exposed group and the control group. This fetal weight recovery could be the result of the observed decrease in sertraline concentrations at the end of gestation.

Earlier research using animal models and cultured cell lines has shown that pro-inflammatory cytokines, along with inflammatory states, can change the expression levels of Abcb1a, Abcb1b, and Oatp4a1 transporters [39,40,41]. Recently, it has been reported that in J774.2 macrophage cells treated with sertraline, the drug exhibits a dual role: it increases pro-inflammatory cytokines (TNF-α, IL-6) via the p38 pathway while reducing others (GM-CSF) through a PI3K-mediated mechanism [42]. This phenomenon may explain the absence of upregulation of *Abcb1a*, *Abcb1b*, and *Oatp4a1* expression in the present study on GD 16, where reduced expression of these transporters was observed in the sertraline-exposed group compared with the unexposed group. This observation is consistent with the observed higher concentrations of sertraline in maternal plasma on GD 16.

Folate transporters FolR1 and RFC mediate folate uptake in the placenta [9]. In the present study, chronic exposure to sertraline significantly increased the expression of *Folr1* on the analyzed GDs. In human hippocampal progenitor cells, sertraline induces glucocorticoid receptor (GR) phosphorylation, which, in turn, promotes its transactivation into the nucleus and the transcription of GR-dependent genes (such as FolR1) by interacting with a glucocorticoid response element (GRE) sequence in the promoter region or by interacting with other transcription factors [43]. Previously, it has been reported that the *FolR1* gene, which lacks a GRE, is indirectly upregulated by the GR agonist dexamethasone in HeLa cells [44]. However, these proposed mechanisms are based on transcriptional regulation and do not demonstrate direct effects at the protein or functional level.

We observed that sertraline treatment increased *Rfc* expression on GD 20 (*p* = 0.046). *Rfc* expression is regulated by several factors, such as the nuclear respiratory factor 1 (NRF-1), vitamin D receptor (VDR), and aryl hydrocarbon receptor (AhR) [15,45,46]. Sertraline regulates the protein expression of VDR [47], which could be one of the mechanisms through which sertraline influences the expression of *Rfc*. However, future studies are needed to explain the molecular mechanisms responsible for the induction of folate transporter expression in the placenta by sertraline.

Folate is a key nutrient for DNA synthesis, one-carbon metabolism, cellular proliferation, and methylation [48]. During pregnancy, these processes become particularly critical due to rapid fetal growth and differentiation, especially the nervous system [49]. In this context, increased expression of Folr1 and Rfc in the syncytiotrophoblast may enhance folate uptake and transport from the maternal circulation to the fetus. Within the framework of this study, *Folr1* and *Rfc* overexpression can be interpreted as an adaptive mechanism aimed at ensuring an adequate folate supply to the fetus, in response to either maternal folate deficiency or placental stress. Given that sertraline has been reported to induce apoptosis mediated by reactive oxygen species in human placental cells and Drosophila larvae [50,51], it is plausible that such placental stress acts as the trigger for this compensatory response.

The Lat1 transporter exchanges large neutral amino acids at the apical membrane of the placenta and acts as a thyroid hormone transporter [52,53]. It has been reported that Lat1 increases markedly as gestation progresses in rats [13], while in the mouse placenta, peak expression occurred on GD 12, decreasing on GDs 16 and 18 [12,54]. Basal expression of *Lat1* decreased from GD 16 to GD 20; however, no significant differences between the exposed and non-exposed placentas were observed on GDs 16 and 20. In contrast, chronic prenatal exposure to paroxetine at doses of 15 and 50 mg/kg decreased *Lat1* gene expression [55]. A recent study has demonstrated that different degrees of prenatal stress in mice result in placental overexpression of *Lat1*, increasing methionine availability and the subsequent global placental DNA hypermethylation and disruption of one-carbon metabolism [56]. The same study revealed that hypothalamic function was altered in fetuses and adults, accompanied by metabolic disorders exclusively in female offspring. However, in the present study, a random pool of female and male placentas was used, as a limited number of placentas for each sex were available. Therefore, further research is needed to determine whether sertraline’s effect is dependent on fetal sex.

To the best of our knowledge, this is the first study evaluating the effect of sertraline on the expression of placental transporters in rats; however, certain limitations in this study warrant emphasis, such as the lack of protein analysis due to the scarce availability of tissue. The use of bulk placental tissue is a limitation of this study; higher-resolution approaches (e.g., laser microdissection or single-cell transcriptomics) would enable more precise discrimination of cell type-specific contributions. The effect of gender on gene expression was not evaluated due to the limited number of samples. The limited number of genes and the scarce number of pathways evaluated also represent major limitations that must be addressed in future studies to provide a more integrative scenario of the fetal and placental effects of gestational sertraline exposure. In addition, the quantification of fetal plasma concentrations should be addressed in subsequent research to strengthen the interpretation of maternal–fetal exposure relationships. Accordingly, the present findings should be interpreted within the context of an exploratory study focused on transcriptional outcomes. Functional, protein-level, and cell-specific analyses will be required to determine the physiological relevance of these observations. Finally, these results must be interpreted cautiously due to the morphological and functional differences between rat and human placentas, limiting direct translation of the findings to humans.

## 4. Materials and Methods

### 4.1. Reagents and Chemicals

The sertraline hydrochloride standard (C_17_H_17_NC_l2_ · HCl) was purchased from Sigma-Aldrich (Sigma-Aldrich, St. Louis, MO, USA), and acetonitrile, methanol, phosphoric acid (H_3_PO_4_), and potassium phosphate monobasic (KH_2_PO_4_) were of high-performance liquid chromatography (HPLC) grade and purchased from Tedia (Tedia, Fairfield, OH, USA).

### 4.2. Animals

Female albino Wistar rats (3 weeks, 30–60 g body weight) were supplied by the Universidad Autonoma de Zacatecas (UAZ) Biotherium, Zacatecas, Mexico. This study was approved by the Research Ethics Committee of the Health Sciences area of the UAZ (CI-UAE-29-2023). The experiments followed the Guidelines for the Use and Care of Research Animals (official Mexican standard NOM-062-ZOO-1999, ARRIVE guidelines, and the Basel declaration, including the 3R concept). Rats were kept in polycarbonate cages at room temperature (25 °C), under controlled relative humidity conditions (60 ± 10%), with a 12 h light/dark cycle, water, and food ad libitum. The animals were provided with a diet composed of 14% protein, 58% carbohydrates, and 19.6% fat (Rodent Lab Chow 5001, Purina^®^, St. Louis, MO, USA).

### 4.3. Treatment

Weaned rats were randomly divided into two groups: 12 were designated as non-exposed (control, receiving vehicle), and 12 were orally administered sertraline. The dose of sertraline administered was 10 mg/kg/day, representing the highest dose at which the lowest fetal reabsorption was observed; this dose is equivalent to 97.25 mg/day in humans [57]. The animals in the control groups received sterile water. The doses were administered orally with an esophageal cannula at a volume of 0.1 mL/10 g of weight.

When the rats reached 12 weeks of age (~250 g), they were mated with 16-week-old males. The presence of a vaginal plug was considered day zero of gestation. The rats were weighed every morning, and pregnancy was confirmed by an exponential weight gain. The analyses were performed on GDs 16 and 20, which represent the last phase of rat development (mostly characterized by somatic growth) [58]. This period is of particular interest for evaluating the expression of placental transporters involved in nutrient transfer.

Six animals per group were used. All groups continued with their respective treatments until the day of euthanasia. For humane euthanasia, animals were deeply anesthetized in an ether chamber, and whole blood was collected by cardiac puncture in accordance with the Mexican Official Standard NOM-033-SAG/ZOO-2014 [59]. All procedures were performed by trained personnel in compliance with ethical guidelines.

### 4.4. Sample Collection

Plasma was separated from whole blood by centrifugation at 12,298× *g* for 10 min and stored at −20 °C until further analysis.

The placentas were collected and washed with cold (4 °C) 1X phosphate-buffered solution (PBS). Tissue pieces (30 mg) were immersed in RNAlater (Sigma-Aldrich) in a 50–50% ratio and stored at 4 °C before processing. The weight of the placenta and fetuses was recorded, and the fetal-to-placental weight ratio (placental efficiency) was calculated. The placental and fetal area was calculated using ImageJ software v2.9.0 (US National Institutes of Health, Bethesda, MD, USA). Tissues were placed on a millimeter-scale grid and photographed. The images were uploaded into the software, and the scale was set by drawing a straight line over the millimeter grid using the straight-line tool to define a known distance. The area of interest was then selected using the polygon selection tool by outlining the contour of the object. Finally, the software provided the area measurements in pixels and real units.

Morphological analysis was carried out to identify any significant congenital alterations in the embryos/fetuses.

### 4.5. Plasma Sample Preparation

Plasma samples were thawed at room temperature, and sertraline was extracted by liquid-phase extraction. A total of 250 µL of 0.1 M NaOH was added to an equal volume of plasma. Subsequently, 1 mL of ethyl acetate was added, agitated for 2 min, and centrifuged at 13,000 rpm for 5 min at 25 °C. The resulting organic layer was separated and evaporated at 50 °C under a nitrogen flow. The sample was reconstituted in a 100 µL mobile phase (A: acetonitrile; B: potassium phosphate monobasic KH_2_PO_4_; C: methanol, in a ratio of 15:35:50 *v*/*v*/*v*).

### 4.6. Chromatography

Samples were analyzed using an HPLC Perkin Elmer Flexar with a UV-Vis LC detector, Binary LC Pump, a C18 column (4.6 × 50 mm and 5 μm (particle size)), and Chromera software v4.1. The mobile phase was isocratic with a constant flow set at 0.6 mL/min. We used a wavelength of 274 nm and an injection volume of 30 µL. The temperature at the injection site was 20 and 25 °C at the column site, and sertraline was retained for 1.6 min.

The analytic method was validated under the criteria of Official Mexican Norm NOM-177 SSA1-2013, which proved to be linear (r ≥ 0. 99 and r^2^ ≥ 0.98), precise (coefficients of variation 2.42% to 10.49 vs. ≤15 for normative criteria), accurate (relative deviation −9.88% to 13.58 vs. ≤15 for normative criteria) and reproducible in a range of 20 to 320 µg/mL.

### 4.7. Gene Expression Analysis

Total RNA was extracted with TRIzol^TM^ (Waltham, MA, USA) reagent from placental homogenates using a Bullet Blender Homogenizer. RNA integrity was verified by 1% agarose gel electrophoresis, and purity and concentration were evaluated using spectrophotometry with a Nanodrop-2000c spectrophotometer (Thermo Fisher Scientific^TM^, Waltham, MA, USA). After DNA-free treatment (Ambion^TM^, Waltham, MA, USA), complementary DNA was synthesized with the GoScript reverse transcription kit (Invitrogen Superscript IV Reverse Transcriptase^TM^, Carlsbad, CA, USA) (oligodT + random primers), quantified with Nanodrop^TM^ (Wilmington, DE, USA), and stored at 4 °C until use. The expression of the six selected transporter genes was evaluated in the two experimental groups and their controls. The assay was carried out in triplicate using quantitative PCR (qPCR) with a StepOne^TM^ (North Shields, UK) instrument and FAM/ZEN/IBFQ probes (IDT™, Iowa, IA, USA). The inter-exonic probes used for gene identification were *Abcb1a* Rn.PT.58.11274852, *Abcb1b* Rn.PT.58.8819022, *Slc7a5* (Lat1) Rn.PT.58.8135252, *Slc19a1* (Rfc) Rn.PT.58.36322795, *Slco4a1* (Oatp4a1) Rn.PT.58.33770631, and *Folr-1* (*Fr-α*) Rn.PT.58.10415588. Glyceraldehyde 3 phosphate dehydrogenase (*Gapdh*) was used as the “housekeeping” gene identified with the probe Rn.PT.39a.11180736.g. 

The system was validated through relative standard curves with efficiencies between 90% and 110%. The amplification conditions were as follows: 95 °C for 20 s, followed by 95 °C for 1 s, and then 60 °C for 20 s for 40 cycles. The relative mRNA levels in each sample were normalized to those of *Gapdh*, whose expression was stable across all experimental groups. Changes in mRNA expression of target genes from the sertraline-treated groups were expressed relative to the control group to obtain the 2-DDCt and relative-quantity (RQ) values.

### 4.8. Statistical Analysis

Data are presented as the mean ± standard deviation (SD) or as medians with minimum and maximum values. Data normality was assessed using the Shapiro–Wilk test. Sertraline concentrations are expressed as the mean ± SD, and comparisons between exposed groups were performed using analysis of variance (ANOVA) followed by Tukey’s post hoc test. Intergroup comparisons of fetal and placental somatometry and gene expression were conducted using the Mann–Whitney U test. Statistical significance was set at *p* < 0.05. All analyses were conducted using SPSS version 30 (IBM Corp., Armonk, NY, USA) and GraphPad Prism version 10.0 (Boston, MA, USA).

## 5. Conclusions

Chronic administration of sertraline does not affect placental somatometry but alters fetal weight and area at mid-gestation. Furthermore, sertraline is associated with changes in the placental gene expression of transporters for hormones, folates, nutrients, and drugs. Further research is needed to better understand the effects of sertraline on the fetus and to contribute to the development of improved pharmacological treatment strategies during pregnancy. These findings provide preliminary transcriptional-level evidence that warrants protein-level functional investigation.

## Data Availability

The original contributions presented in this study are included in the article. Further inquiries can be directed to the corresponding authors.

## Abbreviations

The following abbreviations are used in this manuscript:

SSRI	Selective Serotonin Reuptake Inhibitors
GD	Gestational Day
Gapdh	Glyceraldehyde-3-phosphate dehydrogenase
Abcb1a/1b	ATP-binding cassette transporter
Oatp4a1	Organic anion-transporting polypeptide 4a1
Folr1	Folate receptor α
Rfc	Reduced folate carrier
Lat1	L-type amino acid transporter 1
